# PP24 Incorporation Scenarios For Rare Diseases In Brazil Considering Clinical And Economic Parameters

**DOI:** 10.1017/S0266462325102432

**Published:** 2025-12-29

**Authors:** Letícia Nascimento Guimarães, Ana Luiza Meira Guerra, Carolina Belinassi, Caio Huerta, Gisele Lemes Veiga Araujo, Matheus Costa e Silva, Carolina Guilhon, Leandro dos Santos

## Abstract

**Introduction:**

Rare diseases affect up to 65 per 100,000 individuals, presenting challenges for health technology assessment (HTA). Key issues include evidence quality and incremental cost-utility ratio (ICUR), which influence decisions on incorporating treatments to assist patients with rare diseases in Brazil. This study aimed to evaluate incorporations into the Brazilian Unified Health System (SUS), considering clinical and economic aspects.

**Methods:**

This was a retrospective study conducted from January 2020 to November 2024. Data were collected from the public dossiers available on the Brazilian Ministry of Health (MoH) website, focusing on drugs whose indications were for the treatment of rare diseases. The study included only dossiers with available information on cost-utility analyses that demonstrated incremental effectiveness and incremental cost to the healthcare system; the quality of evidence was evaluated using the GRADE tool. Dossiers with content that did not meet these criteria were excluded from the analysis.

**Results:**

Between January 2020 and November 2024, the Brazilian MoH received 465 technology evaluation requests, with 38 percent (n=176) resulting in decisions of incorporation or expanded use, of which 30 percent (n=53) related to rare diseases. From the analysis of these reports, n=22 matched the inclusion criteria reported above. The ICURs ranged from BRL4,287.24 (USD714.54) to BRL75,938,549.34 (USD12,656,424.90), with an average of BRL4,890,006.53 (USD29,340,039.17); of those, 68 percent exceeded the SUS cost-effectiveness threshold of BRL120,000 (USD20,000) per quality-adjusted life year. Nevertheless, the quality of the evidence was considered low in 70 percent of these evaluations, based on the GRADE tool.

**Conclusions:**

Technologies for rare diseases are often incorporated within the SUS with a high ICUR despite low quality evidence. This is due to the challenging and complex nature of rare diseases, requiring in-depth discussions on adapting HTA methods to these conditions. Developing robust frameworks is essential to ensure equitable access while addressing evidence gaps and decision-making complexities.

